# Halloysite Nanotube-Based Delivery of Pyrazolo[3,4-*d*]pyrimidine Derivatives for Prostate and Bladder Cancer Treatment

**DOI:** 10.3390/pharmaceutics16111428

**Published:** 2024-11-09

**Authors:** Marina Massaro, Rebecca Ciani, Giancarlo Grossi, Gianfranco Cavallaro, Raquel de Melo Barbosa, Marta Falesiedi, Cosimo G. Fortuna, Anna Carbone, Silvia Schenone, Rita Sánchez-Espejo, César Viseras, Riccardo Vago, Serena Riela

**Affiliations:** 1 Dipartimento di Scienze e Tecnologie Biologiche, Chimiche e Farmaceutiche (STEBICEF), Università di Palermo, Viale delle Scienze, Parco d’Orleans II, Ed. 17, 90128 Palermo, Italy; marina.massaro@unipa.it (M.M.); rebecca.ciani@unipa.it (R.C.); 2 Department of Pharmacy, University of Genoa, Viale Benedetto XV, 16132 Genoa, Italy; giancarlo.grossi@unige.it (G.G.); marta.falesiedi@edu.unige.it (M.F.); anna.carbone1@unige.it (A.C.); silvia.schenone@unige.it (S.S.); 3 Dipartimento di Scienze Chimiche (DSC), Università di Catania, Viale Andrea Doria 6, 95125 Catania, Italy; 4 Department of Pharmacy and Pharmaceutical Technology, School of Pharmacy, University of Seville, C/Professor García González 2, 41012 Sevilla, Spain; rdemelo@us.es; 5 Department of Pharmacy and Pharmaceutical Technology, Faculty of Pharmacy, University of Granada, Campus Universitario de Cartuja, 18071 Granada, Spain; ritamsanchez@ugr.es (R.S.-E.); cviseras@ugr.es (C.V.); 6 Andalusian Institute of Earth Sciences, CSIC-UGR, 18100 Armilla, Spain; 7 Istituto San Raffaele (IRCCS), Istituto di Ricerca Urologica, Divisione di Oncologia Sperimentale, 20132 Milano, Italy; vago.riccardo@hsr.it

**Keywords:** pyrazolo[3,4-*d*]pyrimidine derivatives, halloysite, carrier, prostate cancer, bladder cancer, structure-based virtual screening

## Abstract

Background/Objectives: The development of therapies targeting unregulated Src signaling through selective kinase inhibition using small-molecule inhibitors presents a significant challenge for the scientific community. Among these inhibitors, pyrazolo[3,4-*d*]pyrimidine heterocycles have emerged as potent agents; however, their clinical application is hindered by low solubility in water. To overcome this limitation, some carrier systems, such as halloysite nanotubes (HNTs), can be used. Methods: Herein, we report the development of HNT-based nanomaterials as carriers for pyrazolo[3,4-*d*]pyrimidine molecules. To achieve this objective, the clay was modified by two different approaches: supramolecular loading into the HNT lumen and covalent grafting onto the HNT external surface. The resulting nanomaterials were extensively characterized, and their morphology was imaged by high-angle annular dark-field scanning transmission electron microscopy (HAADF-STEM). In addition, the kinetic release of the molecules supramolecularly loaded into the HNTs was also evaluated. QSAR studies were conducted to elucidate the physicochemical and pharmacokinetic properties of these inhibitors, and structure-based virtual screening (SBVS) was performed to analyze their binding poses in protein kinases implicated in cancer. Results: The characterization methods demonstrate successful encapsulation of the drugs and the release properties under physiological conditions. Furthermore, QSAR studies and SBVS provide valuable insights into the physicochemical, pharmacokinetic, and binding properties of these inhibitors, reinforcing their potential efficacy. Conclusions: The cytotoxicity of these halloysite-based nanomaterials, and of pure molecules for comparison, was tested on RT112, UMUC3, and PC3 cancer cell lines, demonstrating their potential as effective agents for prostate and bladder cancer treatment.

## 1. Introduction

Prostate cancer is the second most common cancer in men worldwide, with 1.4 million new cases and 375,000 deaths annually, and bladder cancer is the tenth most commonly diagnosed one, more common in men than in women, with 573,000 new cases and 213,000 deaths annually [1].

Heterocycle compounds and heterocycle fragments, portions of sophisticated macromolecules, are present as active species in a large number of drugs because of their intrinsic versatility and unique physicochemical properties [2]. They bind target enzymes and receptors through supramolecular interactions such as hydrogen bonding, van der Waals forces, pi stacking, hydrophobic forces and so on. Up to now, different heterocycle-based molecules have been synthetized, including tetrahydropyrimidines, pyrrolidone derivatives and so on, which have shown interesting pharmacological activities, especially antitumoral properties on different cancer lines [3,4,5,6,7]. Among the different types of heterocycles, N-heterocycles are the most common structural skeletons in pharmaceuticals on the market [8].

The proto-oncogene c-Src (Src) is a non-receptor tyrosine kinase whose expression and activity are strongly implicated in the development of several human cancers [9,10,11,12,13,14], including bladder [15] and prostate cancer [1,13].

The development of therapies to address unregulated Src signaling with selective inhibition of kinases using small-molecule inhibitors is a challenge for the scientific community [9,16,17,18].

Usually, kinase inhibitors act as competitors with the phosphate-donating ATP, preventing the phosphorylation process. Among the different kinase inhibitors, pyrazolopyrimidine heterocycles, which are bioisosteres of adenine, can mimic ATP, acting as competitors in the signaling pathway [9,19]. In 2013, their clinical use was approved by the FDA [20] and other molecules with a pyrazolopyrimidine skeleton are currently in clinical trials [19,21,22]. For example, Si306, a pyrazolo[3,4-*d*]pyrimidine derivative, has been identified as a potent inhibitor of c-Src tyrosine kinase (K_i_ equal to 0.13 μM) [23], showing good effects on several cancer cell lines [23,24], without affecting healthy cells [25].

However, the further in vivo use of Si306 and other pyrazolo[3,4-*d*]pyrimidine derivatives is hampered by their low solubility in water (for Si306, the solubility in water is 3.7 µg L^−1^) [26]. To overcome this problem, several strategies involving the use of carrier systems have been adopted [23,24]. Halloysite is a clay mineral from the kaolin group that, due to its properties such as biocompatibility, high purity, and bioavailability at a low price, has attracted much attention in recent years [27,28]. Halloysite presents tubular morphology, with an empty lumen, hence the name halloysite nanotubes (HNTs), of a suitable size for encapsulating a large number of molecules [29,30,31].

Recently, we studied the supramolecular interaction with HNTs and Si306 and other pyrazolo[3,4-*d*]pyrimidine derivatives for the development of novel CDK inhibitors. In vitro studies showed that the developed nanomaterials show good antiproliferative activity toward different cell lines [24].

**Figure 1:**
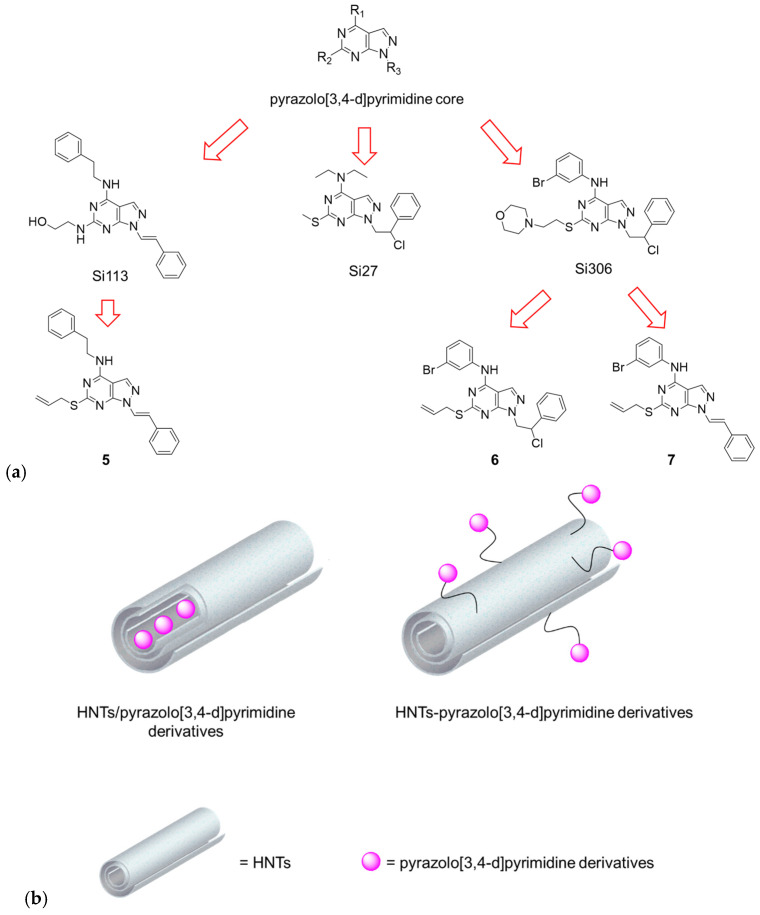
(**a**) Pyrazolo[3,4-*d*]pyrimidine derivatives studied; and (**b**) cartoon representation of HNT-based pyrazolo[3,4-*d*]pyrimidine nanomaterials.

Herein, following our previous study, we report the design and development of a series of halloysite/pyrazolo[3,4-*d*]pyrimidine derivative nanomaterials as potential agents for prostate and bladder cancer treatment. The primary objective of this work is both to expand the scope of the pyrazolo[3,4-*d*]pyrimidine-based molecules for cancer treatment and, encouraged by the good results previously obtained, to develop novel nanomaterials that could possess enhanced activity. To achieve this goal, Si306 and Si113 molecules were ad hoc modified with allyl groups to obtain three novel pyrazolo[3,4-*d*]pyrimidine derivatives (compounds **5, 6** and **7**) (Figure 1a) bearing suitable functional groups to be grafted onto the HNT external surface. Since the introduction of novel functionalities could impair the biological activities of the molecules, preliminary in vitro assays on three distinct human bladder (RT112 and UMUC3) and prostate (PC3) cancer cell lines were performed. To better understand the different biological activities of the synthetized pyrazolo[3,4-*d*]pyrimidine derivatives, some in silico studies were carried out. In particular, quantitative structure–activity relationship (QSAR) studies were performed to provide key information on the physicochemical and pharmacokinetic properties of the inhibitors. Moreover, structure-based virtual screening (SBVS) was conducted to analyze the binding poses of the synthetized molecules in the catalytic pockets of the possible protein kinases involved in human cancer [32]. All the results were compared to the pristine compounds Si306 and Si113, and with Si27, which had never been investigated for these types of tumors (Figure 1a).

Afterward, they were combined with HNTs by covalent grafting onto the HNT external surface by a MW-mediated thiol–ene reaction. To compare the biological effects of the covalently linked molecules on HNTs with the supramolecular nanomaterials, Si113, Si306 and Si27 were also loaded into the HNT lumen. The obtained nanomaterials were extensively characterized, and their morphology was investigated by high-angle annular dark-field scanning transmission electron microscopy (HAADF-STEM). The kinetic release of Si27 from the HNT lumen was also investigated in a medium mimicking the physiological condition. Finally, their cytotoxicity was studied on the RT112, UMUC3 and PC3 cancer cell lines as well.

## 2. Materials and Methods

### 2.1. Chemistry

MW-assisted syntheses were performed with a CEM DISCOVER monomode system CEM Corporation, Matthews, NC, USA) in a closed vessel.

The FT-IR spectra (KBr) were acquired with an Agilent Technologies Cary 630 FT-IR spectrometer (Agilent Technologies, Santa Clara, CA, USA). 

Thermogravimetric analysis was carried out on Q5000 apparatus (TA Instruments, New Castle, DE, USA).

UV–vis measurements were carried out using a Beckmann DU 650 spectrometer (Beckman Coulter, Inc., Brea, CA, USA).

DLS measurements were performed with a Malvern Zetasizer Nano ZS instrument ((Malvern Instruments, London, UK), fitted with a 632 nm laser at a fixed scattering angle of 173°.

TEM images were acquired by means of an FEI Titan G2 60–300 ultra-high-resolution transmission electron microscope (FEI, Lausanne, Switzerland) coupled with analytical electron microscopy (AEM) performed with a SUPER X silicon drift windowless energy dispersive X-ray spectroscopy (XEDS) detector. The AEM spectra were saved in mode STEM (scanning transmission electron microscopy) with an HAADF (high-angle annular dark field) detector.

The HNTs used in this study were obtained from Merck (Milan, Italy) and used as received.

All the commercially available chemicals were used as purchased. CH_2_Cl_2_ was dried over calcium hydride. Anhydrous reactions were run under a positive pressure of dry N_2_ or argon. TLC was performed using Merck (Milan, Italy) TLC plates silica gel 60 F254. Chromatographic purifications were performed on columns packed with Merk 60 silica gel, 200–425 mesh. The ^1^H NMR and ^13^C NMR spectra were recorded on a Brucker Avance DPX400 (Billerica, MA, USA) (at 400 MHz for ^1^H and 100 MHz for ^13^C) in DMSO-d_6_ as the solvent. The chemical shifts (δ) were expressed in parts per million (ppm) relative to tetramethylsilane (TMS), which was used as the internal standard. Data are shown as the following: chemical shift, multiplicity (s = singlet, d = doublet, t = triplet, q = quartet, quint = quintet, sx = sextet, dd = doublet of doublets, tt = triple of triplets, bs = broad singlet), coupling constant (*J*) in Hertz (Hz) and integration. The elemental analysis of C, H, N, and S was determined using a Thermo Scientific Flash 2000 and the results were within ±0.4% of the theoretical value. All the target compounds possessed a purity of ≥95%, as verified by elemental analysis.

Pyrazolo[3,4-*d*]pyrimidin-4-ol **1**, Si113, Si306, Si27, HNTs-SH, HNTs/Si113 and HNTs/Si306 were synthesized by us earlier [24,26,33,34,35].

### 2.2. Synthesis of 6-(Allylthio)-1-(2-hydroxy-2-phenylethyl)-1H-pyrazolo[3,4-d]pyrimidin-4-ol (***2***)

Pyridine (0.3 mL), potassium carbonate (0.22 g, 1.6 mmol, 0.46 eq) and allyl bromide (0.35 mL, 4.05 mmol, 1.2 eq) were added to a suspension of pyrazolo[3,4-*d*]pyrimidin-4-ol **1** (1.0 g, 3.5 mmol, 1 eq) in 40 mL of acetone. This mixture was stirred at room temperature for 4 h. Then, the solvent was evaporated under reduced pressure and the crude was collected by adding diethyl ether and recrystallized from ethyl acetate/diethyl ether 1:1. Yield: 67%. Mp: 180–181 °C. ^1^H NMR (400 MHz, DMSO-d_6_) δ: 3.77 (d, *J* = 7.0 Hz, 2H), 4.27 (dd, *J* = 13.5, 5.8 Hz, 1H, CH_2_N), 4.37 (dd, *J* = 13.5, 7.7 Hz, 1H, CH_2_N), 5.04 (t, *J* = 6.7 Hz, 1H, CHO), 5.10–5.13 (m, 1H), 5.31 (dd, *J* = 17.0, 1.5 Hz, 1H), 5.86–5.96 (m, 1H), 5.60 (br s, 1H, OH), 7.17–7.27 (m, 5H, Ar), 7.89 (s, 1H, H-3), 12.28 (br s, 1H, OH). ^13^C NMR (101 MHz, DMSO-d_6_) δ: 33.09, 54.65, 71.67, 103.35, 126.48, 127.92, 128.58, 133.95, 134.77, 143.08, 158.60, 159.60. Anal. calcd. for C_16_H_16_N_4_O_2_S: C 58.52; H 4.91; N 17,06; S 9.76; found C 58.15; H 4.74; N 16.80; S 9.56.

### 2.3. Synthesis of 6-(Allylthio)-4-chloro-1-(2-chloro-2-phenylethyl)-1H-pyrazolo[3,4-d]pyrimidine (***3***)

The Vilsmeier complex, previously prepared from POCl_3_ (1.2 mL, 8.0 mmol, 1 eq) and anhydrous dimethylformamide (DMF) (1.1 mL, 8.0 mmol, 1 eq), was added to a suspension of **2** (0.26 g, 0.8 mmol, 1 eq) in anhydrous CHCl_3_ (15 mL). The mixture was refluxed for 12 h. The solution was washed with cold water, dried (Na_2_SO_4_), filtered and concentrated under reduced pressure. The crude oil was purified by column chromatography (silica gel 200–425 mesh), using a mixture of petroleum ether (bp 40–60 °C)/diethyl ether (9:1) as the eluent. The obtained oil was recrystallized by adding a mixture of petroleum ether (bp 40–60 °C)/diethyl ether (9:1). Yield: 60%. Mp: 79–80 °C. ^1^H NMR (400 MHz, DMSO-d_6_) δ: 3.82–3.93 (m, 2H), 4.86 (dd, *J* = 14.4, 5.9 Hz, 1H, CH_2_N), 5.00 (dd, *J* = 14.4, 8.5 Hz, 1H, CH_2_N), 5.15 (d, *J* = 10.0 Hz, 1H), 5.37 (d, *J* = 16.9 Hz, 1H), 5.63 (t, *J* = 7.2 Hz, 1H, CHO), 5.92–6.02 (m, 1H), 7.31–7.33 (m, 3H, Ar), 7.46 (d, *J* = 6.5 Hz, 2H, Ar), 8.36 (s, 1H, H-3). ^13^C NMR (101 MHz, DMSO-d_6_) δ: 33.93, 53.81, 60.80, 110.54, 119.06, 128.04, 129.24, 129.62, 133.75, 133.81, 138.20, 153.73, 154.53, 168.61. Anal. calcd. for C_16_H_14_Cl_2_N_4_S: C 52.61; H 3.86; N 15.34; S 8.,78; found C 52.95; H 3.81; N 15.30; S 8.56.

### 2.4. Synthesis of 6-(Allylthio)-1-(2-chloro-2-phenylethyl)-N-phenethyl-1H-pyrazolo[3,4-d]pyrimidin-4-amine (***4***)

Phenethylamine (0.51 mL, 4.05 mmol, 4.0 eq) was added to a solution of **3** (0.37 g, 1.01 mmol, 1 eq) in anhydrous toluene (10 mL). The mixture was stirred for 14 h at room temperature. The solvent was concentrated under reduced pressure and the crude oil was purified by column chromatography (silica gel 200–425 mesh) using at first a mixture of petroleum ether (bp 40–60 °C)/diethyl ether (1:1) and then diethyl ether as the eluents. The obtained colorless oil was recrystallized by adding petroleum ether (bp 40–60 °C)/diethyl ether (1:1). Yield: 58%. Mp: 103–104 °C. ^1^H NMR (400 MHz, DMSO-d_6_) δ: 2.86 (t, *J* = 7.5 Hz, 2H, CH_2_Ph), 3.60–365 (m, 2H, CH_2_NH), 3.75–3.84 (m, 2H), 4.68 (dd, *J* = 14.3, 6.1 Hz, 1H, CH_2_N), 4.82 (dd, *J* = 14.3, 8.6 Hz, 1H, CH_2_N), 5.06–5.09 (m, 1H), 5.28 (dd, *J* = 16.9, 1.7 Hz, 1H), 5.59 (dd, *J* = 8.6, 6.0 Hz, 1H, CHO), 5.94–6.04 (1H, m), 7.15–7.28 (m, 5H, Ar), 7.30–7.36 (m, 3H, Ar), 7.45–7.47 (m, 2H, Ar), 7.98 (s, 1H, H-3), 8.43 (t, *J* = 5.5 Hz, 1H, NH). ^13^C NMR (101 MHz, DMSO-d_6_) δ: 33.62, 35.34, 42.24, 53.05, 61.07, 98.66, 117.78, 126.75, 128.07, 128.93, 129.22, 129.50, 132.98, 135.33, 138.57, 139.85, 154.37, 155.86, 168.11. Anal. calcd. for C_24_H_24_ClN_5_S: C 64.06; H 5.38; N 15.56; S 7.13; found C 63.81; H 5.07; N 15.71; S 6.90.

### 2.5. Synthesis of 6-(Allylthio)-N-phenethyl-1-styryl-1H-pyrazolo[3,4-d]pyrimidin-4-amine (***5***)

To a solution of **4** (0.52 g, 1.15 mmol, 1 eq) in ethanol 95° (20 mL), a solution of sodium hydroxide (0.3 g, 8.40 mmol, 7.3 eq) in water (3 mL) was added. The mixture was refluxed for 5 h and then, after cooling to room temperature, the desired product was collected as a white solid, which was filtered and recrystallized from diethyl ether. Yield: 52%. Mp: 125–126 °C. ^1^H NMR (400 MHz, DMSO-d_6_) δ: 2.90 (t, *J* = 7.4 Hz, 2H), 3.65–3.70 (m, 2H), 3.86 (d, *J* = 6.9 Hz, 2H), 5.06–5.09 (m, 1H), 5.29 (dd, *J* = 17.0, 1.7 Hz, 1H), 5.94–6.04 (m, 1H), 7.16–7.29 (m, 7H, 6Ar + CH), 7.34 (t, *J* = 7.7 Hz, 2H, Ar), 7.55 (d, *J* = 8.7 Hz, 1H, Ar), 7.90 (d, *J* = 14.5 Hz, 1H, CH), 8.18 (s, 1H, H-3), 8.62 (t, *J* = 5.6 Hz, 1H, NH). ^13^C NMR (101 MHz, DMSO-d_6_) δ: 33.73, 35.32, 42.29, 99.58, 116.47, 117.96, 122.63, 126.69, 126.78, 127.89, 128.35, 128.95, 129.24, 129.39, 129.65, 135.02, 135.13, 135.71, 139.78, 153.53, 155.81, 169.16. Anal. calcd. for C_24_H_23_N_5_S: C 69.70; H 5.61; N 16.94; S 7.75; found C 70.02; H 5.38; N 17.01; S 7.40.

### 2.6. Synthesis of 6-(Allylthio)-N-(3-bromophenyl)-1-(2-chloro-2-phenylethyl)-1H-pyrazolo[3,4-d]pyrimidin-4-amine (**6**)

3-Bromoaniline (0.3 mL, 2.6 mmol, 2 eq) was added to a solution of **5** (0.48 g 1.3 mmol, 1 eq) in ethanol absolute (9 mL). The mixture was refluxed for 5 h. The ethanol was evaporated under reduced pressure and the crude oil was purified by column chromatography (silica gel 200–425 mesh) using a mixture of petroleum ether (bp 40–60 °C)/diethyl ether (2:1) as the eluent. The obtained oil was recrystallized by adding petroleum ether (bp 40–60 °C)/diethyl ether (1:1). Yield: 47%. Mp: 115–116 °C. ^1^H NMR (400 MHz, DMSO-d_6_) δ: 3.78–3.88 (m, 2H), 4.76 (dd, *J* = 14.3, 6.0 Hz, 1H, CH_2_N), 4.89 (dd, *J* = 14.3, 8.6 Hz, 1H, CH_2_N), 5.08–5.11 (m, 1H), 5.29 (dd, *J* = 16.9, 1.6 Hz, 1H), 5.63 (dd, *J* = 8.6, 6.0 Hz, 1H, CHO), 5.94–6.04 (m, 1H), 7.24–7.48 (m, 5H, Ar), 7.46–7.48 (m, 2H, Ar), 7.64–7.67 (m, 1H, Ar), 8.15 (t, *J* = 2.0 Hz, 1H, Ar), 8.19 (s, 1H, H-3), 10.19 (bs, 1H, NH). ^13^C NMR (101 MHz, DMSO-d_6_) δ: 33.63, 53.21, 61.02, 99.36, 118.18, 120.14, 121.97, 123.91, 126.60, 128.08, 129.24, 129.53, 131.21, 133.14, 134.70, 138.48, 140.96, 153.56, 154.67, 168.09. Anal. calcd. for C_22_H_19_BrClN_5_S: C 52.76; H 3.82; N 13.98; S 6.40; found C 52.80; H 3.70; N 14.20; S 5.90.

### 2.7. 6-(Allylthio)-N-(3-bromophenyl)-1-styryl-1H-pyrazolo[3,4-d]pyrimidin-4-amine (***7***)

To a solution of **6** (0.26 g, 0.48 mmol, 1 eq) in ethanol 95° (8 mL), a solution of sodium hydroxide (0.15 g, 3.75 mmol, 7.8 eq) in water (2 mL) was added. The mixture was refluxed for 5 h and then, after cooling to room temperature, the ethanol was evaporated under reduced pressure and the desired product was collected as a pale brown solid, which was recrystallized by adding petroleum ether (bp 40–60 °C)/diethyl ether (1:1). Yield: 43%. Mp: 173–174 °C. ^1^H NMR (400 MHz, DMSO-d_6_) δ: 3.88 (d, *J* = 6.7 Hz, 2H), 5.08–5.11 (m, 1H), 5.30 (dd, *J* = 16.9, 1.7 Hz, 1H), 5.94–6.03 (m, 1H), 7.21–7.36 (m, 6H, 5Ar + CH), 7.55–7.57 (m, 2H, Ar), 7.65–7.68 (m, 1H, Ar), 7.95 (d, *J* = 14.6 Hz, 1H, CH), 8.18 (t, *J* = 1.9 Hz, 1H, Ar), 8.37 (s, 1H, H-3), 10.36 (br s, 1H, NH). ^13^C NMR (101 MHz, DMSO- d6) δ: 33.75, 100.37, 117.03, 118.34, 120.22, 122.00, 122.40, 124.01, 126.78, 128.02, 129.39, 131.22, 134.50, 135.04, 135.55, 140.81, 153.56, 153.62, 169.11 Anal. calcd. for C_22_H_18_BrN_5_S: C 56.90; H 3.91; N 15.08; S 6.90; found C 56.77; H 3.84; N 15.20; S 6.56.

### 2.8. Synthesis of HNTs/Si27 Nanomaterial

To an aqueous dispersion (5 mL) of HNTs (0.1 g), 1 mL of a concentrated Si27 solution (MeOH/DMSO (1:1)) (1 × 10^−2^ M) was added. The obtained dispersion was sonicated for 5 min at 25 °C, evacuated for three cycles and left under stirring for 18 h at room temperature. Afterward, the solvent was filtered off, and the resulting powder was washed several times with water and finally dried overnight in an oven at 60 °C. The amount of drug loaded on the HNTs was estimated by TGA.

### 2.9. Synthesis of the HNTs-5, HNTs-6 and HNTs-7 Nanomaterials

HNTs-SH (100 mg) and **5** or **6** or **7** (20 mg) were weighed in an MW test tube provided with a cap in the presence of AIBN as a catalyst. The mixture was inserted in the MW apparatus at 100 °C, under constant stirring, for 1 h. Successively, the solid was washed several times with water and dried at 60 °C under a vacuum.

### 2.10. Kinetic Release

The release of Si27 from the HNTs/Si27 nanomaterial was performed as follows: 20 mg of the sample was dispersed in 1 mL of phosphate buffer pH 7.4 and transferred into a sealed dialysis membrane (Medicell International Ltd., London, UK, MWCO 12–14000 with a diameter of 21.5 mm). Afterward, the membrane was put in a round-bottom flask containing 9 mL of the release medium at 37 °C and stirred. At a fixed time, 1 mL of the release medium was withdrawn and analyzed by UV–vis measurements at λ of 290 nm. To ensure sink conditions, 1 mL of fresh solution was used to replace the collected one. The total amounts of drug released (*F_t_*) were calculated as follows:

(1) $$F_{t}=V_{m}C_{t}+\sum_{i=0}^{t-1}V_{a}C_{i}\;$$

where *V_m_* and *C_t_* are the volume and the concentration of Si27 at time *t*. *V_a_* is the volume of the sample withdrawn and *C_i_* is Si27 concentration at time *i* (*i* < *t*).

### 2.11. Cell Cultures

Human bladder cancer RT112 (grade 2) and UMUC3 (grade 3) and human prostate cancer PC3 (grade 3) cell lines were maintained in RPMI-1640 medium (R0883-Merck Life Science, Darmstadt, Germany), supplemented with 10% fetal bovine serum (FBS Euroclone, catalog number ECS0180L, Pero, MI, Italy) and 1% of penicillin/streptomycin. The cells were maintained at 37 °C in a humidified atmosphere of 5% CO_2_ and passaged using trypsin–EDTA (Sigma-Aldrich, Burlington, MA, USA, catalog number SLCH3365).

### 2.12. Cell Viability Assay

To test the effect of loaded HNTs on cell viability, cells were plated in 96-well plates at a cell density of 5 × 10^3^ cells/well and treated with serial dilutions of drug-loaded HNTs, ranking from 0.2 μM to 10 µM, for 72 h.

At time 0, the medium was replaced with fresh complete medium supplemented with the synthetized nanomaterials. Following 72 h of treatment, an MTT assay was performed according to the manufacturer’s recommendations (Sigma-Aldrich). Briefly, following 72 h of treatment, the cells were washed with PBS, and 100 µL of RPMI supplemented with thiazolyl blue tetrazolium bromide (5 µg mL^−1^) was then added to each well and further cultured for 2 h at 37 °C in CO_2_ incubator. Finally, the medium containing MTT was removed and 100 μL of DMSO was added to each well to dissolve the formazan. The absorbance was recorded at 570 nm using a microplate reader (Spark^®^ 20 M Tecan Trading AG, Männedorf, Switzerland). The percentage of cell viability with respect to control cells treated with only the solvent where the compounds were resuspended was calculated after subtraction of the blank. The IC_50_ values, i.e., the concentration necessary to inhibit 50% of the cell growth, was calculated using a dose–response model, obtained from sigmoidal fitting of the response curves of the percent inhibition versus the concentration of each compound. Each result represents the average value of three different experiments performed in quadruplicate.

### 2.13. Computational Methods

The binding poses in the human tyrosine active sites were generated using FLAP software in the structure-based mode (Software version 2.2.2. on 12 February 2020Molecular Discovery Ltd., Borehamwood, UK; www.moldiscovery.com, accessed on 12 February 2020). The procedure has been extensively described in the Supplementary Materials. The protein binding site was calculated with the FLAP site module in the FLAP software, which is able to reproduce the main cavities of the crystallographic structures. For each ligand, a maximum of 25 conformers were generated to mimic the compound flexibility; the protonation state at a physiological pH of each molecule was assigned to the ionizable residues, as predicted by MoKa [36,37].

The FLAP software was used in the “structure-based” mode; this approach allowed us to generate the binding poses of a ligand in a protein cavity based on the similarity between the GRID fields. The GRID molecular interaction fields were generated through the probes for H (shape), DRY (hydrophobic interactions) N1 (H-bond donor) and O (H-bond acceptor) interactions.

## 3. Results and Discussion

### 3.1. Synthesis of the Pyrazolo[3,4-d] Pyrimidine Derivatives and Study of Their Biological Properties

**Scheme 1:**
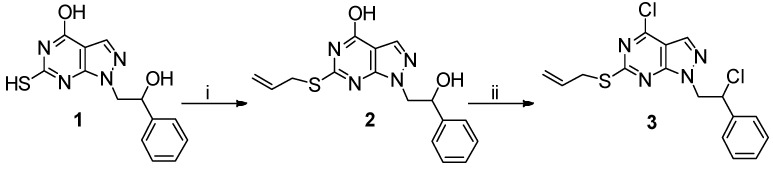
Synthesis of key intermediate **3**. Reagents and conditions: (i) allyl bromide, acetone, K_2_CO_3_, pyridine, r.t., 4 h, 67%; and (ii) DMF an., POCl_3_, CHCl_3_ an., reflux, 12 h, 60%.

**Scheme 2:**
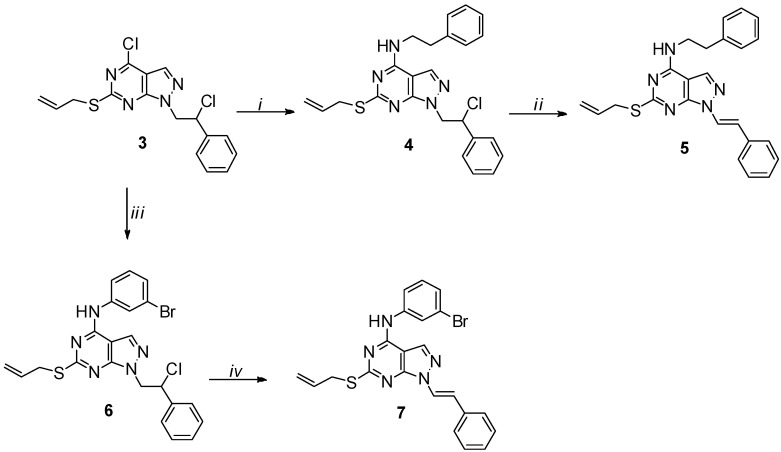
Synthesis of final compounds **5**–**7**. Reagents and reaction conditions: (i) PEA, toluene an., r.t., 14 h, 58%; (ii) EtOH, NaOH, reflux, 5 h, 52%; (iii) 3-bromoaniline, EtOH abs., reflux, 5 h, 47%; and (iv) EtOH, NaOH, reflux, 5 h, 43%.

The novel pyrazolo[3,4-*d*] pyrimidine derivatives obtained in this study were synthetized starting from a pyrazolo[3,4-*d*]pyrimidin-4-ol **1**, as previously reported by some of us (Scheme 1) [35]. In particular, it was reacted with allyl bromide in acetone to give the corresponding C6 thioallyl derivative 2 (67%). This latter compound, by reaction with the Vilsmeier complex (POCl_3_/DMF), afforded the desired dichloro key intermediate **3** in a good yield (60%) (Scheme 1). Derivative **3** was treated with phenethylamine to obtain the 4-phenethylamino derivative **4** (58%), which led to the final compound **5** (Scheme 2) by reaction with sodium hydroxide in ethanol under reflux in a satisfactory yield (52%). Furthermore, the treatment of **3** with 3-bromoaniline gave the desired compound **6** (47%), which, after the reaction with sodium hydroxide in ethanol at reflux, provided its unsaturated analogue **7** (43%) (Scheme 2). All the synthetized molecules were characterized by the ^1^H and ^13^C NMR spectroscopies, as reported in the Experimental Section.

**Figure 2:**
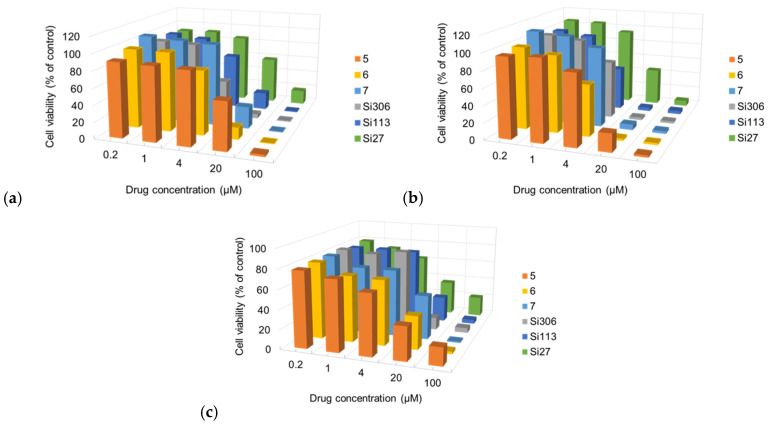
Effect of increasing concentrations of the compounds **5**, **6**, **7** and Si113, Si306, Si27 and Si306 on the RT112 (**a**), UMUC3 (**b**) and PC3 (**c**) cell viability after 72 h of treatment. Each result represents the average value of four different experiments performed in quadruplicate. Cell viability is expressed as a percentage compared to untreated cells.

The biological properties of the synthetized molecules were studied on three distinct human bladder (RT112 and UMUC3) and prostate (PC3) cancer cell lines. For comparison, we also tested, beside the **5**–**7** molecules, their precursors Si113 and Si306, and the Si27 compound, the effects of which were never investigated on these types of tumor cells. The collected data are reported in Figure 2, whereas the viability curves with the relative errors are reported in the Supporting Information. Because of their highly hydrophobic nature, all the compounds were first solubilized in DMSO and then diluted in cell culture medium to minimize the presence of the solvent.

All the compounds exerted a dose-dependent cytotoxic effect on all the tested cell lines. To better compare the effects of different compounds and analogues, the half-maximal inhibitory concentration (IC_50_) was calculated for each compound (Table 1).

Si113 and Si306 were more effective than Si27 in reducing the cell viability in the RT112 and UMUC3 bladder cancer cell lines, while the effect was comparable on the PC3 prostate ones. Compounds **5**, **6** and **7** maintained the capability to consistently reduce the cell viability in a similar manner compared to their precursors, indicating that the modifications did not drastically alter their activity.

To better understand the different biological activities of the pyrazolo[3,4-*d*]pyrimidine derivatives, we performed in silico studies of them with a human tyrosine–protein kinase c-Src (pdb code: 1FMK) involved in both prostatic and bladder tumor [1,13,15,38,39].

**Figure 3:**
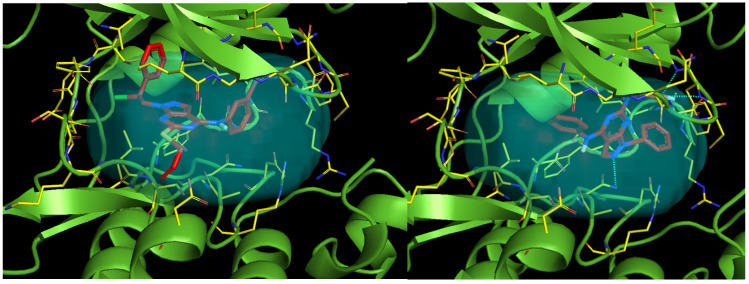
Proposed 3D binding mode inside the active site of human tyrosine, determined by FLAP analysis; in particular, the 3D binding pose in pocket one for compound Si113 on the left and compound **6** on the right side.

A structure-based virtual screening (SBVS), based on the Fingerprints for Ligands and Proteins (FLAP) algorithm, was carried out in an attempt to rationalize the inhibition capabilities of the compounds **5**–**7** and for comparison with the Si113, Si306 and Si27 ones. Furthermore, the binding poses of all the compounds in the catalytic pockets of the human tyrosine–protein kinase were analyzed. FLAP is able to calculate seven pockets of possible interaction, but we decided to use only pocket one (Figure 3) and pocket six (Figure S1) for the virtual screening; in particular, in pocket one, there is the catalytic site and it is located in the center of the kinase region, while in pocket six, there is the co-crystallized Tyr 527 in order to select the closed, autoinhibited conformation.

The following compounds were chosen for screening: the three novel compounds nicknamed **5**, **6** and **7**, and the two compounds Si113 and Si27, which, respectively, show the highest and lowest activity toward the cell lines.

In the Supporting Information, we report the table of the GLOB-SUM calculated by the FLAP program. It is possible to notice that the trend of the GLOB-SUM values seems to respect the trend of the biological activities reported in the present paper; in fact, it can be seen that the compound Si113 is the one with the highest score, while the Si27 has the lowest score, and the three new compounds (**5**, **6** and **7**) are located within this range.

Compound **6** is the one with the highest value among the three unknown compounds, also presenting a 3D pose very similar to that of Si113, as reported in Figure 3.

**Figure 4:**
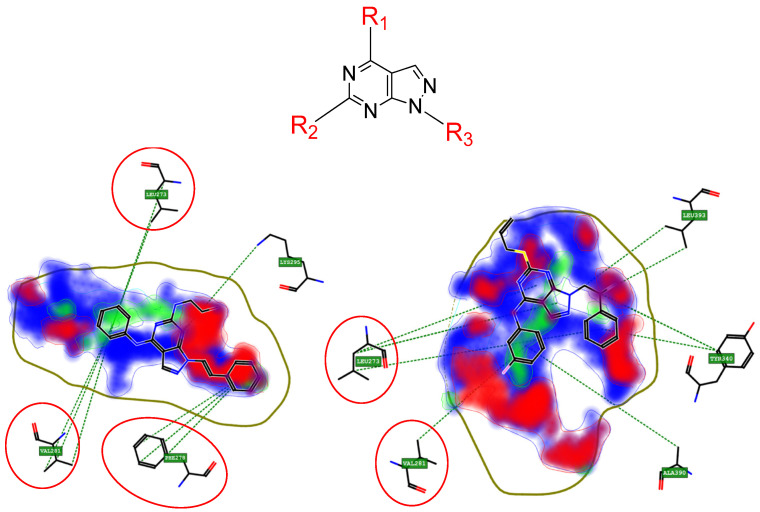
Two-dimensional binding pose in pocket one for compound Si113 on the left and compound **6** on the right side. N1 (Amide NH) probe describes the hydrogen bond donor character of interaction (blu areas in figures). DRY (Hydrophobic) probe describes the hydrophobic character of interaction (green areas in figure). O (Carbonyl Oxygen) probe describes the hydrogen bond acceptor character of interaction (red areas in figure).

It may make sense to compare the 2D residues of compounds **5**, **6** and **7** with those of Si113. In particular, circled in red, we report the residues in common with those involved in the interaction with the Si113; all three ligands show at least two residues in common, as shown in Figure 4.

All the binding poses for the other compounds with pocket one and pocket six are reported in the Supplementary Materials.

But why do they have lower GLOB-SUM scores? One reason could be the absence of interaction with the Lys295 residue, which, as we have seen from the literature, is the one involved in the phosphating process; this lack is, probably, due to the absence of an OH group in the R_2_ (Figure 4) arm of the pyrazolo[3,4-*d*]pyrimidine skeleton.

Comparing the 3D poses of compounds **5**, **6** and **7**, we see how **6** has a different position inside the pocket compared to the others. This difference can also be seen from the 2D depiction, where it is possible to notice the two strong interactions with the “arm” R_3_ of the pyrazole core; specifically, there are two interactions with residues Tyr340 and Leu393 with the aromatic ring and chlorine, where the chlorine replaces the double bond present in the R_3_ arm in the other two compounds.

Another difference lies in the interaction areas involved, the so-called shape of the molecule. From the 2D depiction, it is possible to notice how compound **6** has a different binding area compared to the other two compounds and also larger H-bond donor/acceptor interaction areas (areas in red and blue), while you can see how the green areas (hydrophobic interactions) are equally distributed across the entire compound.

### 3.2. Synthesis, Characterization and Biological Studies of HNTs Hybrid Systems

In order to improve the aqueous solubility of the pyrazolo[3,4-*d*]pyrimidine derivatives and their cellular uptake, they were combined with HNTs by two different approaches: (i) a supramolecular loading of the pyrazolo[3,4-d]pyrimidine derivatives Si113, Si306 and Si27 into the HNT lumen and (ii) covalent grafting of compounds **5**, **6** and **7** onto the HNT external surface.

### 3.3. Loading of Si113, Si306 and Si27 Derivatives

The supramolecular HNTs/pyrazolo[3,4-*d*]pyrimidine derivative nanomaterials were obtained by supramolecular loading of the Si113, Si306 and Si27 compounds, previously synthetized, into the HNT lumen, following a procedure reported elsewhere (Figure 1) [24,26,33,34]. After work-up, the amount of pyrazolo[3,4-*d*]pyrimidine derivatives loaded inside the HNT lumen was quantified by TGA, as large as of ca. 4 wt% for HNTs/Si113 and HNTs/Si306, respectively, whereas it was ca. 2 wt% in the case of HNTs/Si27.

### 3.4. Covalent Grafting

**Scheme 3:**
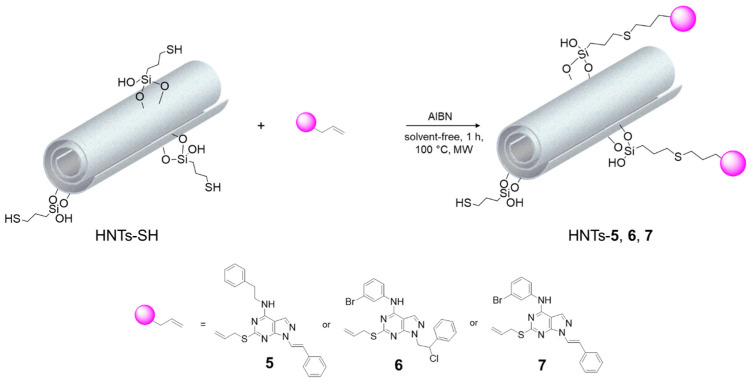
Synthesis of pyrazolo[3,4-*d*] pyrimidine derivative-based halloysite nanomaterials.

The **5**, **6** and **7** compounds were grafted onto the HNT external surface by a microwave-assisted AIBN-catalyzed thiol–ene reaction, using as a reactant a thiol-modified HNT in solvent-free conditions at 100 °C, according to Scheme 3. After work-up, the loading of **5** and **6** onto the HNTs was ca. 2 wt%, whilst the loading of **7** was as large as 1 wt%, as estimated by TGA, which correspond to a degree of functionalization of 0.05, 0.04 and 0.02 mmol g^−1^ for HNTs-**5**, HNTs-**6** and HNTs-**7**, respectively. On the basis of the stoichiometric ratio between the -SH groups on the HNTs-SH surface (degree of functionalization 0.22 mmol g^−1^) and the pyrazolo[3,4-*d*] pyrimidine derivatives grafted, it is possible to conclude that not all the -SH groups were involved in the thiol–ene reaction, probably due to steric hindrance.

**Figure 5:**
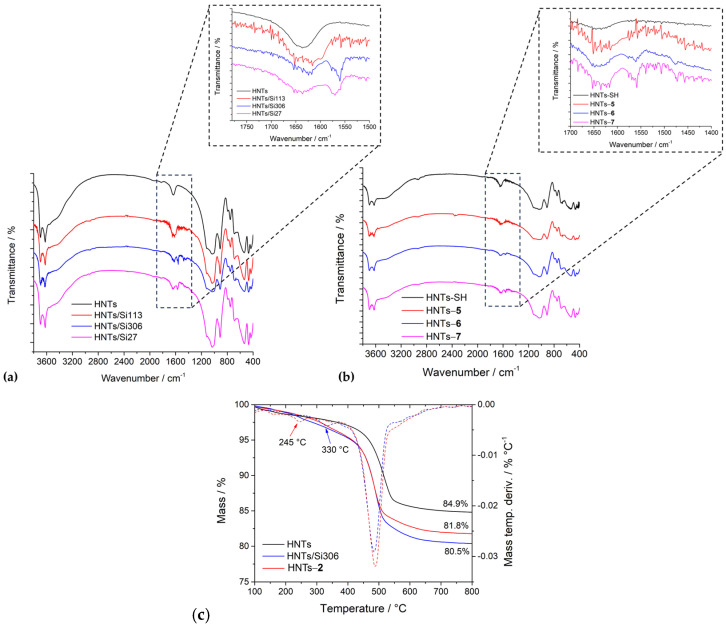
(**a**,**b**) FT-IR spectra of the synthetized nanomaterials; and (**c**) thermogravimetric curves (straight line) and their derivatives (dots line) of the HNTs, HNTs/Si306 and HNTs-**6** nanomaterials.

All the synthetized nanomaterials were characterized by several physico-chemical techniques. In Figure 5a,b, the FT-IR spectra of the nanomaterials synthetized and the ones of the pristine HNTs and HNTs-SH precursors are reported. As it is possible to observe, after the pyrazolo[3,4-*d*] pyrimidine derivatives’ loading and grafting, beside the typical HNTs vibration bands some additional peaks are present as well. In particular, there are clearly observable the bands at ca. 1580, 1560 and 1477 cm^−1^ due to the C–C, C–N and C–N stretching vibrations of the phenyl and pyrazolo[3,4-*d*] pyrimidine groups of the organic molecules.

Figure 5c shows the TGA curves of the HNTs/Si306 and HNTs-**6** nanomaterials and that of pristine HNTs for comparison. As one can see, besides the typical mass losses of halloysite arising from the expulsion of the interlayer water molecules of the HNTs (ca. 550 °C), additional mass losses attributable to the degradation and volatilization of organic matter are present in the TGA curves of the nanomaterials. These mass losses were evidenced by the presence of different peaks, centered at ca. 245 and 330 °C, for HNTs-**6** and HNTs/Si306, respectively, in the differential thermogravimetric curves, further confirming the presence of the molecules on the HNTs. Furthermore, it is very interesting to observe from the TGA data that the degradation temperature of the organic moieties is dependent on the investigated system. In particular, the HNTs/Si306 nanomaterial shows a higher degradation temperature (330 °C) of the organic matter than the one of HNTs-**6** (245 °C) due to the presence of the Si306 compound in the HNT lumen, while **6** is covalently attached to the HNT external surface. Similar conclusions can be drawn for the other synthetized nanomaterials.

The aqueous mobility of all the synthetized nanomaterials was investigated by dynamic light scattering measurements (DLS) and the obtained results are reported in Table 2. The supramolecular loading of Si306, Si113 and Si27 into the halloysite lumen, as expected, did not lead to any variation in the Z-average size of the nanomaterials in comparison to the pristine HNTs, further confirming the presence of the organic molecules inside the halloysite lumen. On the contrary, the covalent grafting of the pyrazolo[3,4-*d*] pyrimidine derivatives onto the external surface of the HNTs slows the aqueous diffusion of the HNTs, as shown by the increased values of the Z-average size of the nanomaterials in comparison to that of the pristine HNTs. These findings could be explained considering the occurrence of hydrophobic interactions between the organic moieties covalently linked onto the outer surface of the halloysite. Similar observations were found for HNTs with a selective modification of the external surface by exploiting both covalent and supramolecular interactions [40]. As concerns the surface charge, all the nanomaterials synthetized show more positive ζ-potential values than that of the pristine HNTs, indicating that a modification of the tubes’ surfaces occurs.

**Figure 6:**
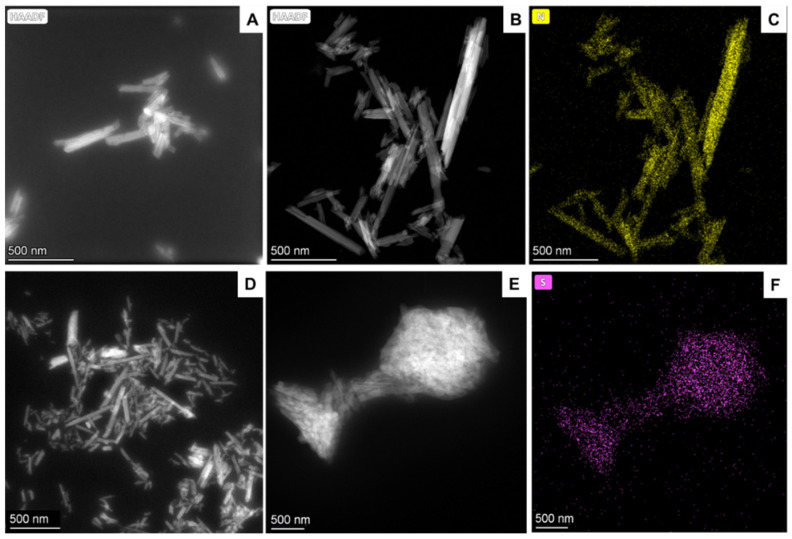
HAADF/STEM images of (**A**) HNTs/Si27, (**B**,**C**) HNTs-**6** and (**D**–**F**) HNTs-**7** nanomaterials.

The morphology of the different nanomaterials was imaged by TEM and high-angle annular dark-field scanning transmission electron microscopy (HAADF-STEM). Similar to that already reported for HNTs/Si306 and HNTs/Si113 [24], the morphology of HNTs/Si27 is pretty similar to that of pristine HNTs (Figure 6A). Also, in this case, indeed, the typical tubular structure of halloysite is clearly observable, where the lumen of the tubes is barely discernible, confirming the interaction of the Si27 compound with the HNT inner surface. Conversely, a different morphology was observed when a covalent modification of the HNT external surface occurs. TEM images (Figure 6B–F) of the HNTs-**6** and HNTs-**7** showed the presence of HNT compact structures, where the tubes are “glued” together, probably due to the presence of pyrazolo[3,4-*d*]pyrimidine derivatives on the surface that can establish for each other favorable attraction interactions such as, for example, π–π interactions.

An important feature that should be considered when the application of the nanomaterial as drug carrier systems for anticancer purposes is envisaged, is the kinetic release of the drug from the carrier. In the present study, since the HNTs-**5**, HNTs-**6** and HNTs-**7** nanomaterials present the drug covalently linked to the HNT external surface, it could be useful to investigate the kinetic release of the pyrazolo[3,4-*d*] pyrimidine derivatives supramolecularly loaded into the halloysite lumen. Previous studies showed that the kinetic release of Si306 and Si113 from the HNT lumen is very slow in physiological media, where ca. 5 wt% and 15 wt% of the total amount of Si306 and Si113 loaded, respectively, was released within 48 h. The different amount of drug released was explained by taking into account the slighter enhanced solubility of Si113 compared to Si306 in water (solubility of 126.5 μg L^−1^ and 3.7 μg L^−1^ for Si113 and Si306, respectively). These results highlighted that the HNTs could effectively act as nanocontainers for the slow and sustained release of pyrazolo[3,4-*d*] pyrimidine derivatives.

**Figure 7:**
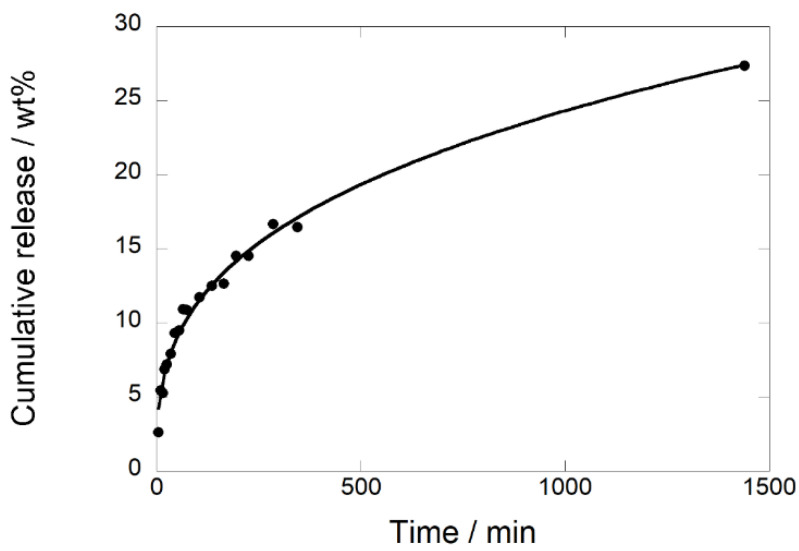
Kinetic release of Si27 from HNTs/Si27 nanomaterial in phosphate buffer pH 7.4 at 37 °C.

In light of these results, herein, we investigate, to expand the scope of applicability of pyrazolo[3,4-*d*] pyrimidine derivatives halloysite-based carrier systems, the kinetic release of Si27 from HNTs/Si27 nanomaterial. This was evaluated by the dialysis bag method, using conditions designed to mimic physiological conditions (phosphate buffer pH 7.4, 37 °C) and the obtained kinetic data are shown in Figure 7. In these conditions, ca. 25 wt% of the total amount of Si27 loaded was released after 24 h.

The kinetic data were analyzed by different mathematical models to obtain information about the release mode. Two models, namely the first-order and Power Fit, were applied. The obtained fits revealed that the release of Si27 from HNTs/Si27 follows the Power Fit model (*k* = 2.5 ± 0.1 min^−1^, *n* = 0.33 ± 0.01, R^2^ = 0.9877), indicating a diffusion from the HNT lumen. Since the *n* value is lower than 0.45, the release of Si27 from HNTs/Si27 corresponds to a Fickian diffusion.

### 3.5. Cytotoxic Effect on Cancer Cell Lines

**Figure 8:**
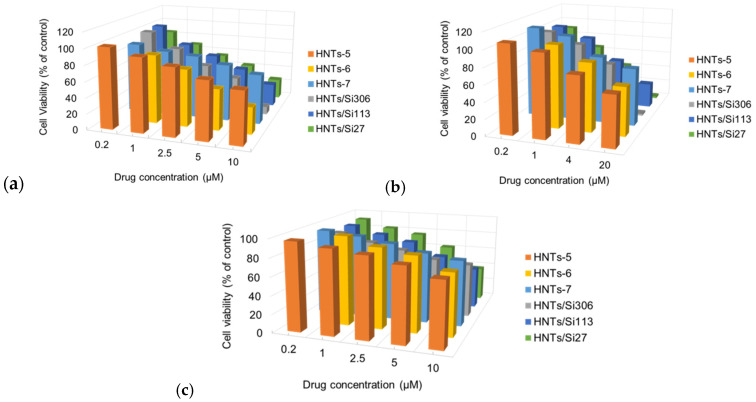
Effect of increasing concentrations of loaded HNTs on (**a**) RT112, (**b**) UMUC3 and (**c**) PC3 cell viability after 72 h of treatment. Each result represents the average value of four different experiments performed in quadruplicate. Cell viability is expressed as percentage compared to untreated cells.

A cell viability assay was carried out to evaluate the effect of the HNT-based pyrazolo[3,4-*d*]pyrimidine derivatives on the same cancer cell lines as described above. RT112, UMUC3 and PC3 cancer cells were treated with increasing concentrations of HNT-based nanomaterials and the cell viability was evaluated after 72 h. The collected data are reported in Figure 8, whereas the viability curves with relative errors are reported in the Supporting Information.

Most HNT-based nanomaterials exerted a dose-dependent cytotoxic effect on the tested cell lines. The IC_50_ could be calculated for some loaded HNTs and it was compared to the pristine compounds (Table 3).

The results demonstrate that HNTs can effectively deliver drugs without compromising their activity. The IC_50_ of the loaded HNTs is comparable to pristine compounds or even reduced in several cases. The RT112 cell line was shown to be the most responsive to the treatments. HNTs/Si113, HNTs/Si306, HNTs-**2**, and HNTs/Si27 have been found to have an effect on cell viability at lower dosages with respect to pristine drugs. The HNTs had no toxic effects per se and greatly improved the water solubility of the system and allowed dispersion of the nanocomposite in the culture media and reduced the negative effects of organic solvents like DMSO, opening up the possibility of future use in in vivo experiments. Although these findings are very promising, the IC_50_ values found are lower than that obtained when treating RT112 cells with a HNTs/epirubicin nanomaterial that was ca. 0.18 ± 0.03 mM. The different behavior could be ascribed to (i) the greater antitumoral efficacy and (ii) solubility in physiological conditions of epirubicin in comparison to pyrazolo[3,4-d]pyrimidine derivatives; and (iii) the enhanced bioavailability of epirubicin in HNTs/epirubicin nanomaterial, since this drug is preferentially adsorbed onto the clay external surface [41]; conversely, the pyrazolo[3,4-*d*]pyrimidine derivatives used in this study are loaded into the HNT lumen.

To investigate if the lack of cytotoxic activity at concentrations lower than 10 µM of some loaded HNTs (HNTs-**5**, HNTs-**6** and HNTs-**7**) in all the cell lines was due to a limited dispersion of the drugs, we increased the concentration of DMSO to 10%, but again, the IC_50_ was not detectable.

## 4. Conclusions

In this study, we successfully developed a series of halloysite/pyrazolo[3,4-*d*]pyrimidine derivative nanomaterials aimed at enhancing the treatment of prostate and bladder cancer. By leveraging the unique properties of halloysite nanotubes (HNTs), including their tubular morphology, biocompatibility, and cost-effective bioavailability, we addressed the significant challenge of low solubility faced by pyrazolo[3,4-*d*]pyrimidine derivatives. Three novel derivatives (compounds **5**, **6**, and **7**) were synthesized and incorporated into HNTs through supramolecular loading and covalent grafting. The resulting nanomaterials were extensively characterized, demonstrating successful encapsulation and release properties under physiological conditions. Furthermore, QSAR studies and structure-based virtual screening (SBVS) provided valuable insights into the physicochemical, pharmacokinetic, and binding properties of these inhibitors, reinforcing their potential efficacy. Biological evaluations on human bladder (RT112, UMUC3) and prostate (PC3) cancer cell lines revealed significant antiproliferative activity, confirming the promise of these nanomaterials as effective therapeutic agents. The innovative combination of pyrazolo[3,4-*d*]pyrimidine derivatives with halloysite nanotubes offers a compelling strategy for overcoming solubility challenges and enhancing drug delivery and efficacy in cancer treatment. Future research should focus on further optimizing these nanomaterials, exploring additional derivatives, and conducting in vivo studies to validate their therapeutic potential and safety profile. The integration of halloysite nanotubes into drug delivery systems represents a promising frontier in the fight against cancer, potentially leading to more effective and targeted therapies.

## Figures and Tables

**Table 1:** IC_50_ of Si113, Si306, Si27 and compounds **5**–**7** on the RT112, UMUC3 and PC3 cell lines after 72 h of treatment.

Drug	RT112	UMUC3	PC3
**5**	25.0 ± 5.0	9.9 ± 0.1	9.3 ± 2.1
**6**	10.4 ± 2.9	5.0 ± 1.3	10.7 ± 1.5
**7**	13.6 ± 4.3	9.0 ± 1.7	16.3 ± 2.1
Si306	3.6 ± 1.2	7.2 ± 1.1	8.3 ± 0.6
Si113	8.0 ± 0.7	4.1 ± 1.6	9.5 ± 0.9
Si27	26.7 ± 2.9	16.8 ± 1.6	10.0 ± 3.0

**Table 2:** Average size, polydispersity index and ζ-potential values of the synthetized nanomaterials. Reported are the mean ± SD values of three independent experiments run in triplicate.

Nanomaterial	Z-Average Size/nm	PDI	ζ-Potential/mV
HNTs	295 ± 16	0.300	−19.0
HNTs/Si306	330 ± 20	0.481	−16.9
HNTs/Si113	299 ± 32	0.350	−12.6
HNTs/Si27	273 ± 8	0.368	−15.4
HNTs-**5**	654 ± 80	0.678	−14.7
HNTs-**6**	1026 ± 170	0.744	−13.3
HNTs-**7**	1110 ± 70	0.895	−12.3

**Table 3:** IC_50_ derived from the loaded HNT dose–response curves on RT112, UMUC3 and PC3 cell lines after 72 h of treatment. The IC_50_ from three different experiments is reported as the mean ± SE.

Drug	RT112	UMUC3	PC3
HNTs-**5**	>10	>10	>10
HNTs-**6**	2.8 ± 0.8	>10	>10
HNTs-**7**	>10	>10	>10
HNTs/Si306	4.3 ± 0.6	3.4 ± 1.8	>10
HNTs/Si113	4.6 ± 0.4	6.3 ± 1.5	8.5 ± 1.6
HNTs/Si27	3.4 ± 0.8	6.5 ± 0.9	7.3 ± 0.5

## Data Availability

Dataset available on request from the authors.

## Supplementary Materials

The following supporting information can be downloaded at https://www.mdpi.com/article/10.3390/pharmaceutics16111428/s1, FLAP values, referring to pocket 1 of the 1FMK domain, sorted in descending GLOB-SUM basis, 2D and 3D binding poses for compound Si27 in pocket 1 of 1FMK human tyrosine, 2D and 3D binding poses for compound **5** in pocket 1 of 1FMK human tyrosine, 2D and 3D binding poses for compound **7** in pocket 1 of 1FMK human tyrosine.
