# Supplementary material for: Halloysite Nanotube-Based Delivery of Pyrazolo[3,4-d]pyrimidine Derivatives for Prostate and Bladder Cancer Treatment
Source: Pharmaceutics. 2024 Nov 9;16(11):1428. doi: 10.3390/pharmaceutics16111428 (PMC11597611; doi:10.3390/pharmaceutics16111428)

Supporting Information

Halloysite Nanotube-Based Delivery of Pyrazolo[3,4-d]pyrimidine Derivatives for Prostate and Bladder Cancer Treatment

Marina Massaro,[a] Rebecca Ciani,[a] Giancarlo Grossi,[b] Gianfranco Cavallaro,[c] Raquel de Melo Barbosa,[d] Marta Falesiedi,[b] Cosimo Fortuna,[c,*] Anna Carbone,[b] Silvia Schenone,[b] Rita Sánchez-Espejo,[e] César Viseras,[e,f] Serena Riela,[c*] Riccardo Vago[h]

[a]Dipartimento di Scienze e Tecnologie Biologiche, Chimiche e Farmaceutiche (STEBICEF), Università di Palermo, Viale delle Scienze, Parco d'Orleans II, Ed. 17, 90128 Palermo, Italy.

[b]Department of Pharmacy, University of Genoa, Viale Benedetto XV, 16132 Genoa, Italy

[c]Dipartimento di Scienze Chimiche (DSC), Università di Catania, Viale Andrea Doria 6, 95125, Catania, Italy. E-mail: cgfortu@unict.it; serena.riela@unict.it.

[d]Department of Pharmacy and Pharmaceutical Technology, School of Pharmacy, University of Seville, C/Professor García González 2, 41012 Sevilla, Spain

[e]Department of Pharmacy and Pharmaceutical Technology, Faculty of Pharmacy, University of Granada, Campus Universitario de Cartuja, 18071 Granada, Spain.

[f]Andalusian Institute of Earth Sciences, CSIC-UGR, 18100 Armilla, Granada, Spain.

[g]Istituto San Raffaele (IRCCS), Istituto di Ricerca Urologica, Divisione di Oncologia Sperimentale, 20132-Milano, Italy.

Table of Content

1. FLAP values, referring to pocket 1 of 1FMK domain, sorted in descending Glob-Sum basis…..S3
2. 2D and 3D binding poses for compound Si27 in Pocket 1 of 1FMK Human Tyrosine……..…..S4
3. 2D and 3D binding poses for compound **5** in Pocket 1 of 1FMK Human Tyrosine……………..S5
4. 2D and 3D binding poses for compound **7** in Pocket 1 of 1FMK Human Tyrosine……………..S6
5. ^{1}H and ^{13}C NMR spectra of compounds **5**, **6** and **7**……………………………………..…..S7

Table S1. FLAP values, referring to pocket 1 of 1FMK domain, sorted in descending Glob-Sum basis.

Candidate	Glob-Sum	Glob-Prod	Distance	N1	DRY	O	H
**1 - Si113**	2.16774	0.608979	7.79769	0.284485	0.953554	0.396015	0.973588
**6j**	2.12149	0.596632	7.81771	0.290332	0.960731	0.408333	0.981567
**6k**	2.10269	0.673985	7.85324	0.242921	0.780598	0.435985	0.987113
**5j**	2.08439	0.652942	8.23728	0.274005	0.714719	0.36898	0.980924
**6h**	2.08308	0.630503	7.81621	0.293355	0.804542	0.454062	0.979039
**5 - Draft 3**	2.0779	0.567582	8.50188	0.144195	1.18648	0.345103	0.960443
**6b**	2.07122	0.621875	8.04619	0.21915	0.862799	0.441266	0.978664
**4h**	2.05155	0.643793	7.71706	0.301797	0.869639	0.38842	0.98954
**4g**	2.04729	0.627894	8.1925	0.306065	0.681032	0.507509	0.985192
**2 - Si305**	2.04298	0.557679	8.83631	0.123173	0.986066	0.350862	0.945704
**1j**	2.033	0.614356	8.4374	0.136985	0.941744	0.377673	0.980021
**4c**	2.02557	0.592573	8.14582	0.245808	0.83523	0.333972	0.979303
**4i**	2.02205	0.612996	8.35895	0.2531	0.782315	0.406408	0.983218
**6c**	2.02024	0.567265	8.1434	0.224763	0.810937	0.451962	0.984082
**5e**	1.99667	0.555468	8.43817	0.23401	0.934693	0.295296	0.984216
**6 - Draft 2**	1.99663	0.563058	8.47325	0.118984	1.07414	0.405486	0.971667
**1g**	1.98914	0.521329	8.64937	0.146472	1.0607	0.391027	0.97169
**1i**	1.98295	0.561906	8.96609	0.095071	0.925402	0.319418	0.993304
**4 - Draft 1**	1.96866	0.548433	8.91487	0.129432	1.03616	0.383186	0.962567
**6i**	1.96695	0.6402	8.06312	0.285255	0.692442	0.504446	0.981928
**5l**	1.96255	0.575682	8.61834	0.213447	0.754649	0.35899	0.978873
**6a**	1.95723	0.623026	8.0312	0.202783	0.807159	0.51684	0.992008
**5i**	1.95502	0.599068	8.21288	0.246896	0.827227	0.416297	0.974104
**5k**	1.95255	0.560546	8.56111	0.180953	0.835572	0.490549	0.991516
**3 - Si27**	1.95032	0.731967	10.4031	2.00E-06	0.905975	0.364153	0.977549
**1d**	1.93305	0.50785	9.1224	0.143256	0.964312	0.287215	0.978405
**7 - Roscovitine**	1.92826	0.599424	8.10266	0.249238	0.747153	0.436705	0.977626
**6d**	1.91836	0.555291	8.48183	0.215005	0.811421	0.415539	0.978744
**4e**	1.9151	0.582186	8.71529	0.219163	0.731673	0.408533	0.990415
**4f**	1.91412	0.613588	8.27572	0.305478	0.700486	0.424036	0.989141
**6e**	1.91013	0.58186	8.2608	0.250042	0.851876	0.370957	0.995197
**4b**	1.9034	0.611814	8.57508	0.252329	0.733841	0.328811	0.981006
**1c**	1.88017	0.581045	8.62245	0.216198	0.83479	0.367905	0.979441
**5f**	1.87973	0.527715	9.01486	0.20805	0.808971	0.352473	0.988004
**4d**	1.87822	0.577604	8.65804	0.219925	0.729497	0.390424	0.987315
**6g**	1.86666	0.602679	8.37322	0.233774	0.636934	0.516301	0.983486
**1h**	1.86654	0.513551	9.20207	0.129591	0.823073	0.360984	0.981002
**6f**	1.86607	0.563487	8.19925	0.221735	0.809697	0.467878	0.975138
**1f**	1.86153	0.519622	9.36293	0.121954	0.910116	0.278523	0.980392
**4a**	1.85926	0.509357	9.01729	0.284018	0.712027	0.41275	0.990486
**1b**	1.85813	0.553011	9.07123	0.212597	0.770308	0.280713	0.993363
**5h**	1.85257	0.540649	9.13498	0.192073	0.755662	0.373686	0.986272
**5b**	1.84305	0.536979	9.73535	0.202894	0.697366	0.237934	0.993541
**5c**	1.8421	0.542115	9.50079	0.198204	0.806059	0.303353	0.988197
**1e**	1.84058	0.544487	9.14042	0.1905	0.928684	0.278853	0.986877
**1k**	1.81323	0.577547	9.18065	0.130548	0.695619	0.421831	0.986871
**5d**	1.7748	0.58469	9.21002	0.219191	0.69646	0.347939	0.986942
**5g**	1.77133	0.532655	9.60737	0.14277	0.675224	0.276017	0.98847
**5a**	1.75244	0.510259	9.62491	0.181644	0.664363	0.250841	0.983549
**1a**	1.75032	0.566061	9.00668	0.243423	0.761898	0.37124	0.980624

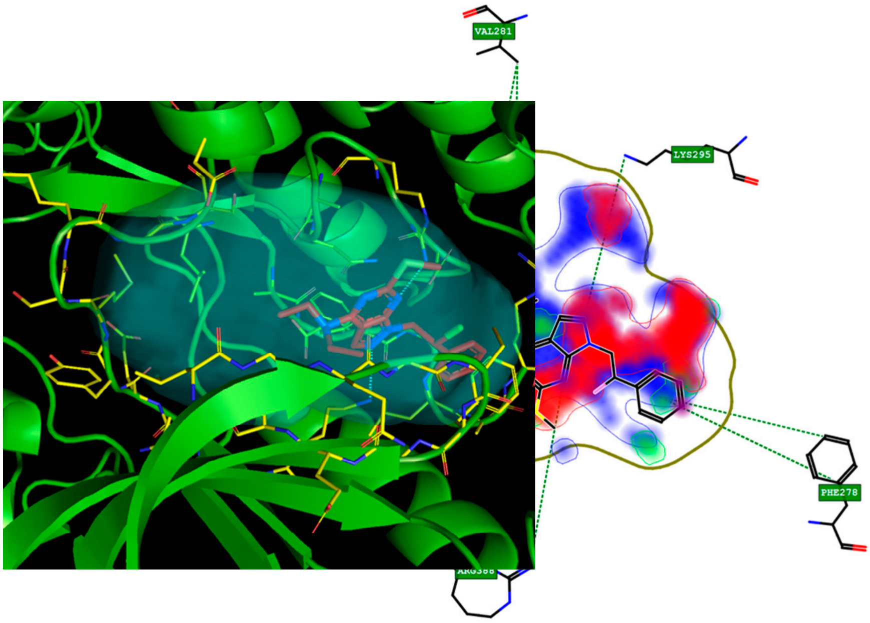

Figure S1. 2D and 3D binding poses for compound Si27 in Pocket 1 of 1FMK Human Tyrosine.

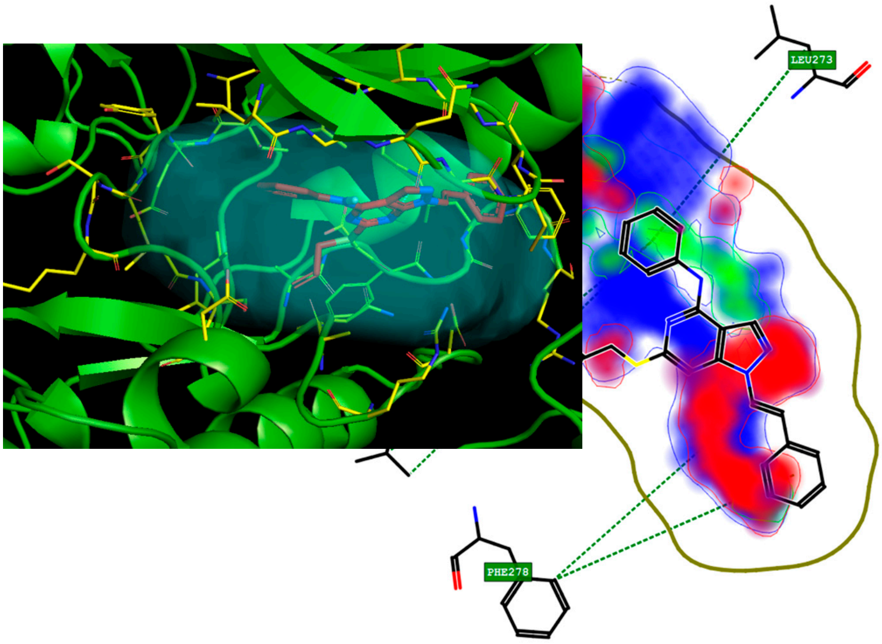

Figure S2. 2D and 3D binding poses for compound **5** in Pocket 1 of 1FMK Human Tyrosine.

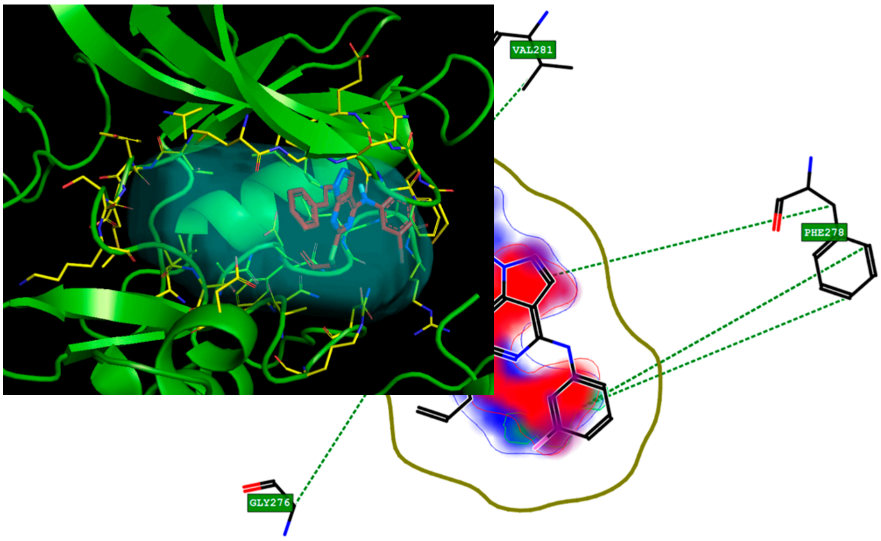

Figure S3. 2D and 3D binding poses for compound **7** in Pocket 1 of 1FMK Human Tyrosine.

Compound 5

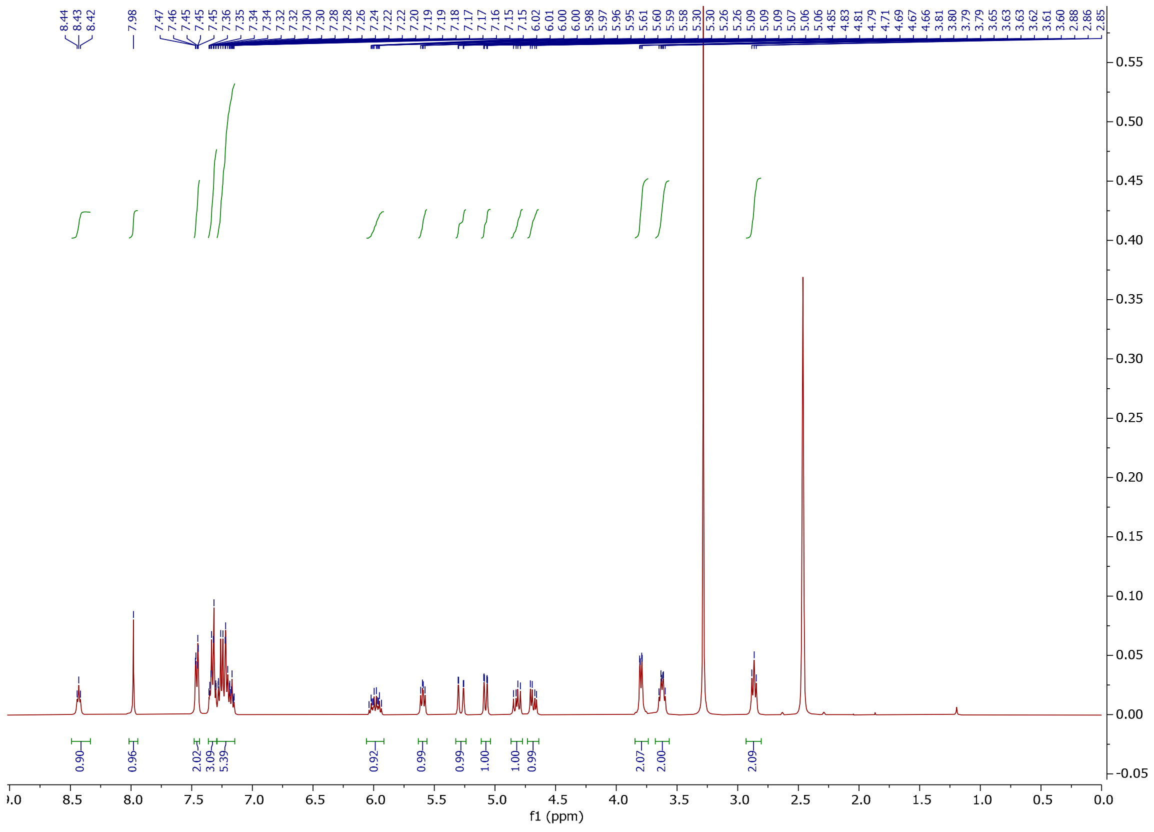

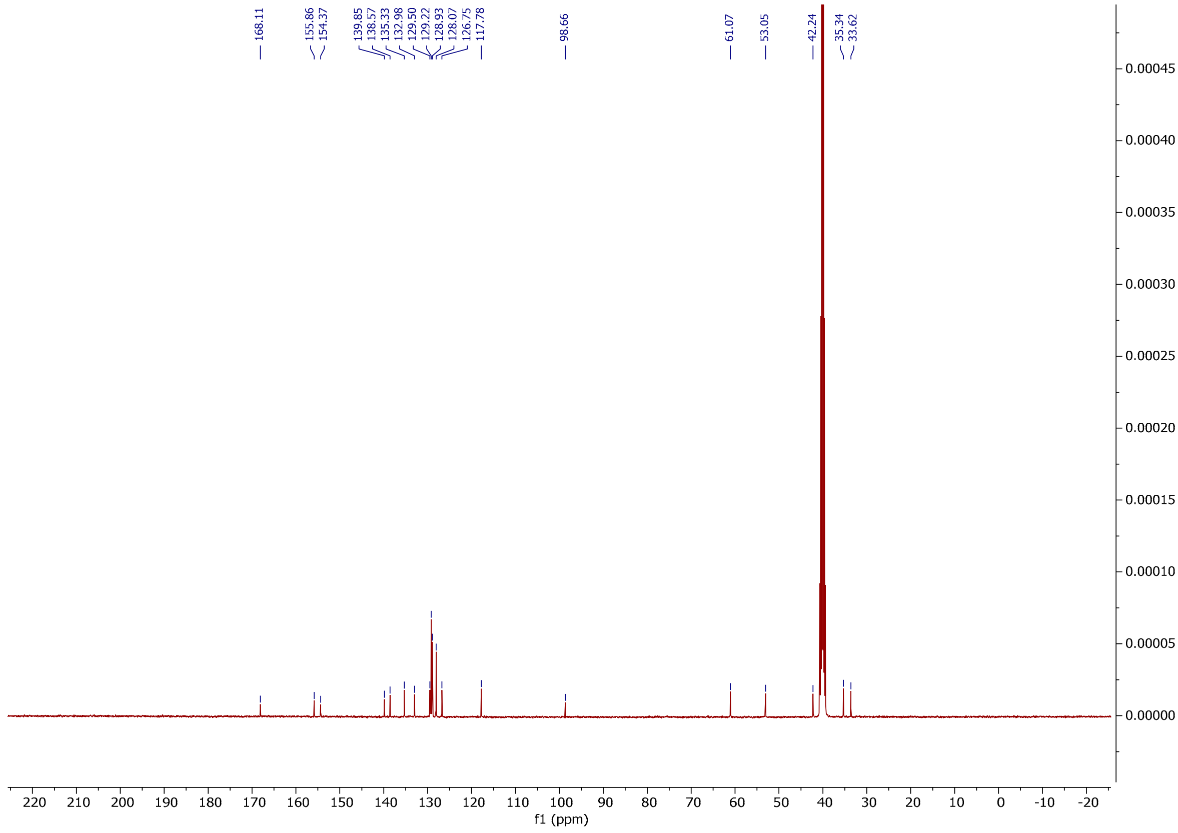

Compound 6

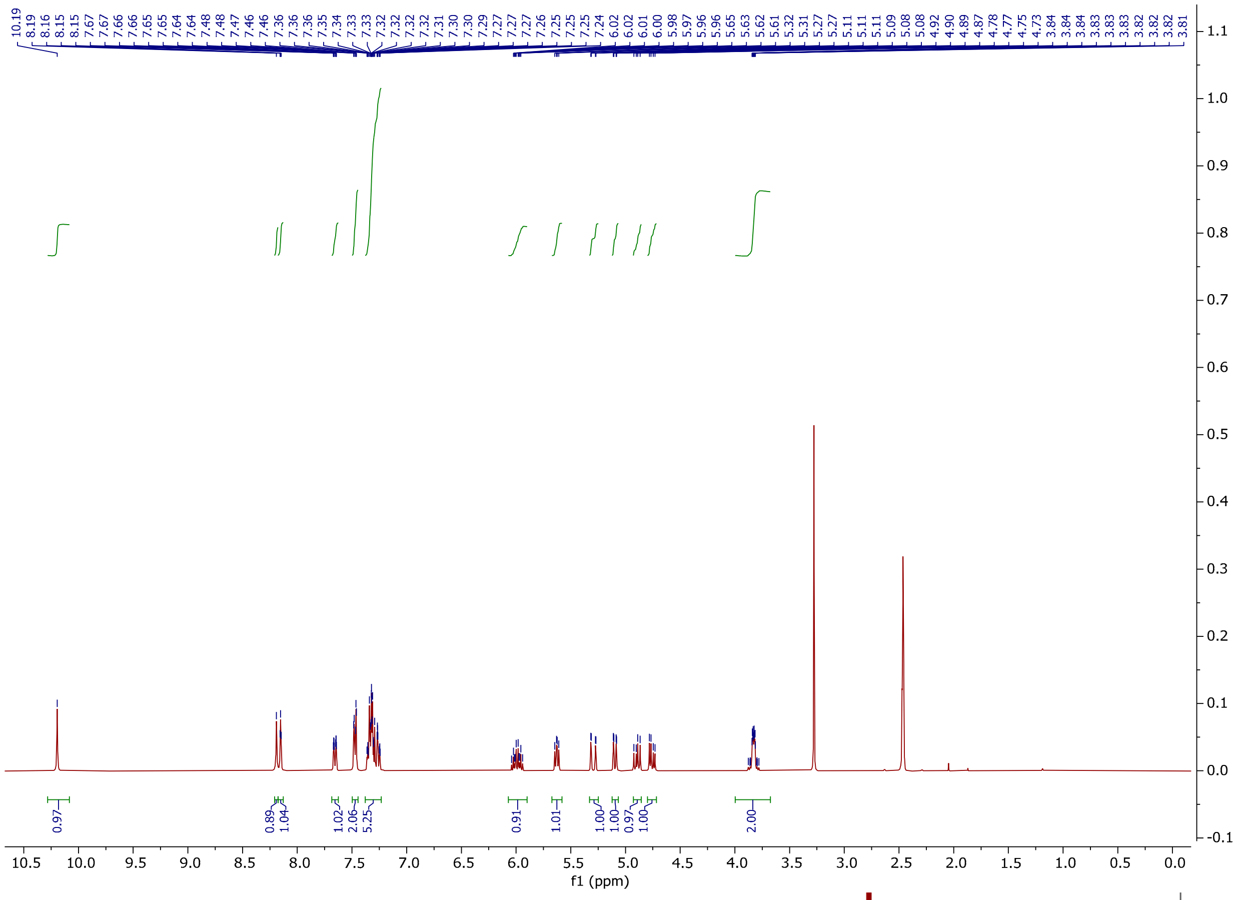

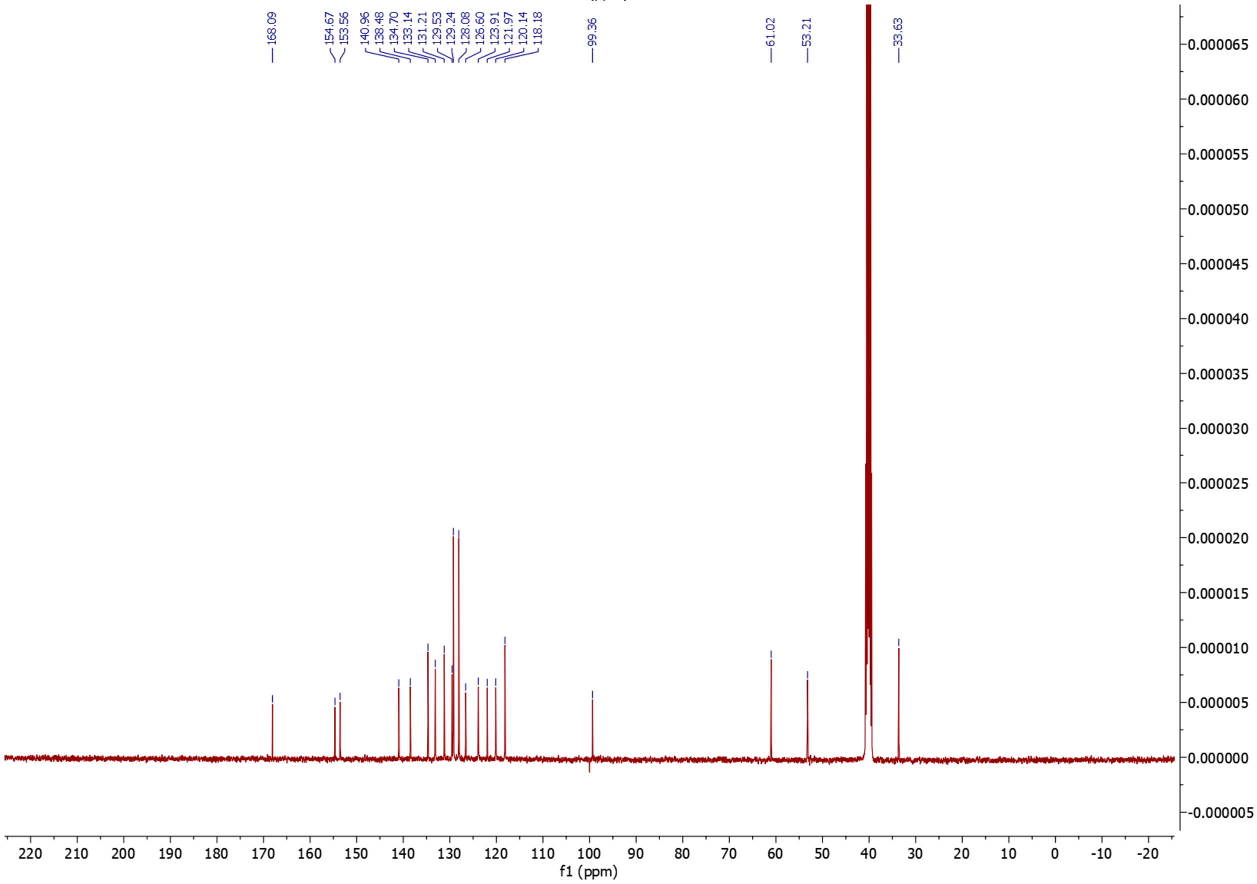

Compound 7

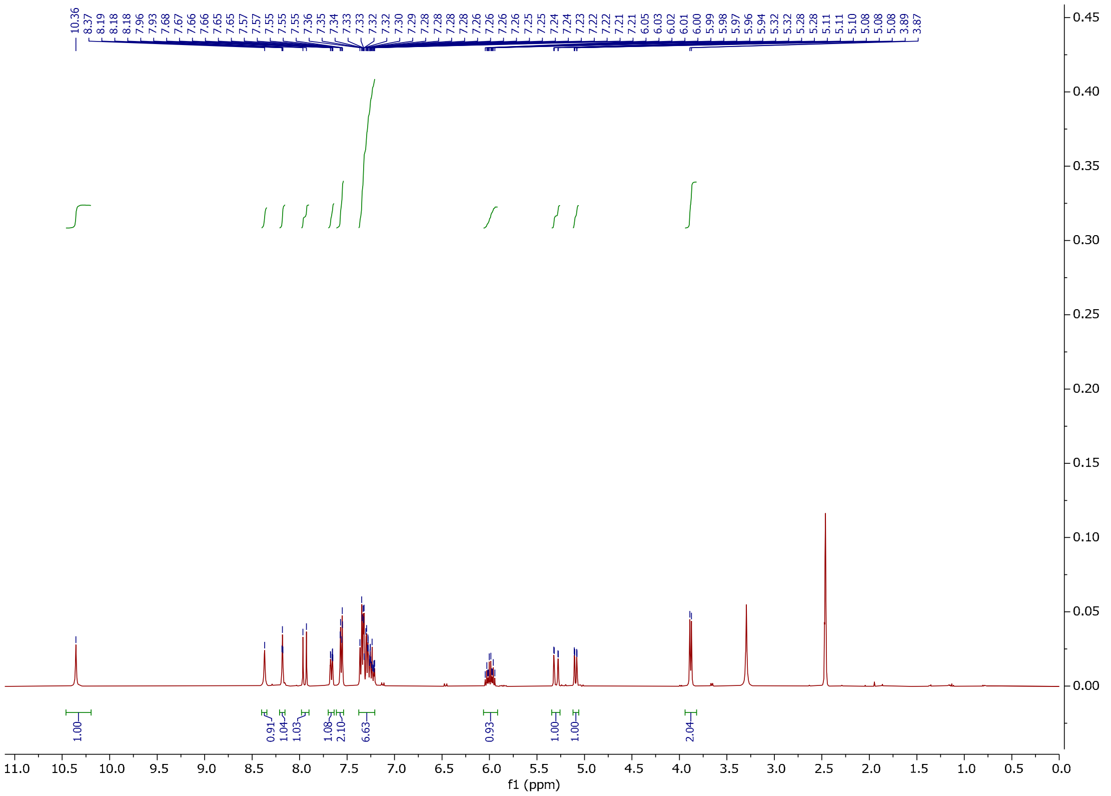

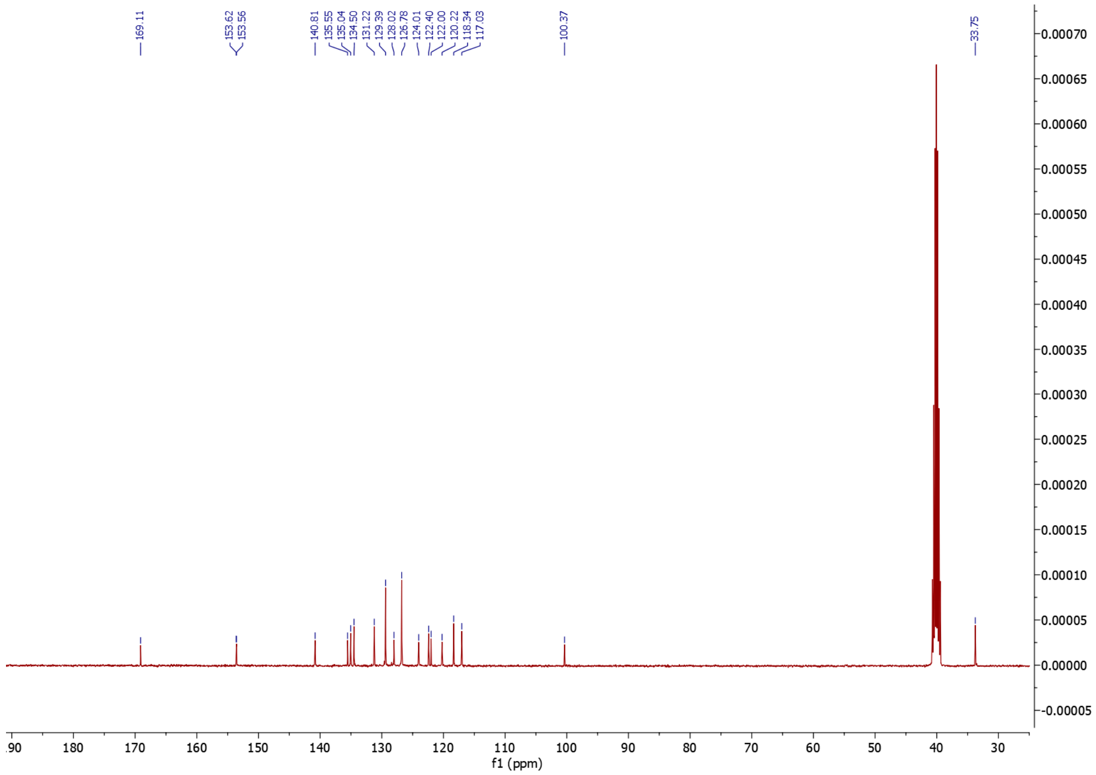

Supplement: Supplementary file 1 [file pharmaceutics-16-01428-s001.zip › pharmaceutics-3209074-supplementary.pdf]
